# Effect of blade parameters on radial turbine rotor aerodynamics

**DOI:** 10.1038/s41598-025-33442-4

**Published:** 2026-01-14

**Authors:** Mostafa Abdo Fawaz, Ahmed Farid Ayad Hassan, Mohammed Shaheen, M. F. Al-samieh

**Affiliations:** 1 Mechanical Design and Production Department, Military Technical College, Cairo, Egypt; 2 Aerospace Department, Military Technical College, Cairo, Egypt

**Keywords:** Energy science and technology, Engineering

## Abstract

Radial turbines play a vital role in turbochargers and compact power systems, where efficiency and size optimization are crucial. However, the combined aerodynamic effects of key rotor geometric features–namely blade angle distribution, thickness profile, and blade count–have not been comprehensively examined. This work presents a unified CFD-based methodology to assess how coordinated changes in these parameters influence turbine performance. A validated numerical model of a reference rotor was employed to systematically vary each design factor and evaluate its impact on efficiency and reduced mass flow. The investigation demonstrates that carefully optimized geometric adjustments can enhance flow uniformity, minimize secondary losses, and improve overall energy conversion. The study establishes clear performance trends supported by detailed flow-field analysis and provides design-oriented correlations that can guide future optimization of radial turbine rotors for high-efficiency operation.

## Introduction

Radial turbines are widely used in turbochargers and compact power-generation systems for their efficient energy conversion, mechanical robustness, and adaptability to varying operating conditions. Their performance directly impacts engine efficiency, transient response, and emissions, making aerodynamic optimization increasingly important.

**Fig. 1:**
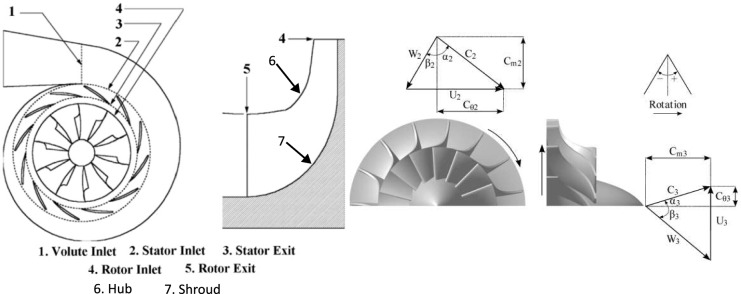
Schematic of a radial turbine stage. Modified from Noughabi, A.K.; Sammak, S., Applied Sciences, 2018, 8, 2207, licensed under CC BY 4.0^14^.

A typical radial turbine stage consists of a stationary nozzle (stator) that accelerates and directs gas onto a rotating rotor. The rotor blades, extending from hub to shroud, extract energy while guiding radial flow inward. Figure 1 shows the main components, including stator, rotor, hub, shroud, and inlet/outlet planes. Each blade is defined by its *camberline*, *thickness distribution* (*t*), and inlet/outlet angles ($\beta _2$, $\beta _3$), which govern the flow path and orientation through the stage.

Rotor performance is strongly influenced by blade count, thickness profile, and the variation of blade angles. Blade angle distribution significantly affects flow turning and aerodynamic losses. Abdelhamid^1^ demonstrated that a $10^\circ$ increase in inlet angle can increase output power by roughly 3%, while Hatazawa et al.^2^ showed that varying outlet angles by $\pm 15^\circ$ could alter efficiency by 4–7%. Mezher et al.^3^ observed efficiency reductions exceeding 7% when blade angles were poorly optimized. Although these studies underscore the importance of angle distribution, they often examine it independently, without addressing interactions with thickness or blade number.

Blade thickness influences both aerodynamic performance and structural durability. Reducing blade thickness can improve efficiency by decreasing blockage and wake losses, while excessively thin blades risk structural failure. Schobeiri et al.^4^ reported that lowering blade thickness by 20–30% increased efficiency by 2–3%, whereas thickness beyond 1.2 times the baseline resulted in a 5–6% loss. Other investigations^5,6^ confirm that shaping thickness profiles to match flow requirements mitigates boundary-layer separation and secondary vortices, demonstrating that uniform scaling is often insufficient.

Blade count also affects internal flow and overall performance. Adding blades improves flow guidance and reduces cross-passage deviations but increases viscous losses. Kim et al.^7^ observed efficiency gains of 1.5–2% when increasing blade count from 8 to 12, while Alford et al.^8^ noted efficiency reductions of up to 3% for blade counts above 14 due to excessive solidity. These findings highlight the trade-off between improved flow alignment and increased blockage or friction.

Recent developments in optimization and surrogate-assisted design have expanded the ability to enhance radial turbine performance. Mehrnia et al.^9^ achieved 3–5% efficiency improvements using metaheuristic algorithms with CFD^10^ by refining inlet angles, and Vitale et al.^11^ employed adjoint-based optimization for multistage designs. Wang et al.^12^ combined neural networks with multiobjective algorithms to explore larger design spaces efficiently. However, these approaches typically examine one or two geometric factors at a time, leaving the combined effects of blade angle, thickness, and count–particularly for small-scale turbocharger rotors–largely unexplored.

This study addresses this gap by conducting a systematic CFD analysis of the BorgWarner K03/05 radial turbine rotor^13^. Multiple geometric configurations are evaluated to quantify how variations in blade angle ($-90\%$ to $+100\%$ relative to baseline), blade thickness (0.1–$1.3\times$ baseline), and blade count (7–14 blades) affect flow patterns, pressure distribution, and total-to-static efficiency. The results provide detailed insight into the sensitivity of radial turbine performance to these key parameters and establish a foundation for future design optimization.

## Methodology

**Fig. 2:**
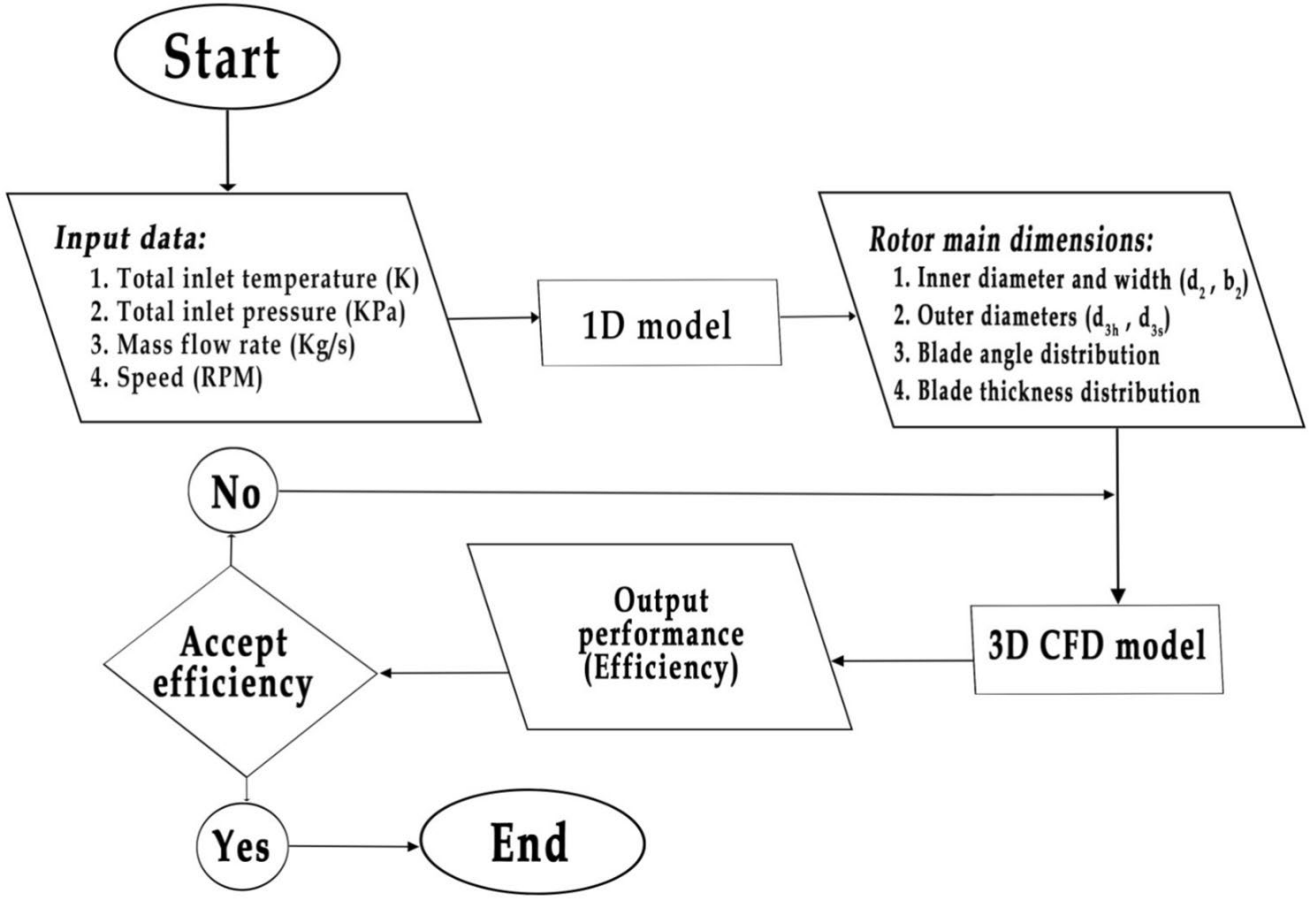
The rotor design tool flowchart.

Fawaz et al.^15^ developed a simulation-based design framework for radial turbine rotors that integrates one-dimensional (1D) modeling with three-dimensional CFD simulations. Figure 2 shows the overall workflow of the rotor design and evaluation tool. The process starts with the 1D meanline model, which takes design specifications–mass flow rate, total pressure ratio, rotational speed, and inlet total temperature–to compute preliminary rotor dimensions and velocity triangles. These outputs define the initial geometric parameters, including hub and shroud radii, blade height, and flow angles. The resulting geometry is then used to build the 3D CFD model for detailed flow-field simulations predicting aerodynamic performance. The flowchart thus illustrates the integration of 1D design and 3D CFD validation, linking input conditions, geometry generation, and performance assessment within a unified framework.

In this study, rotor geometries were generated using a previously developed automated modeling tool^15^, enabling rapid creation of 3D designs by systematically varying selected parameters. When the blade angle distribution, thickness profile, or blade count was modified, the tool automatically produced the corresponding 3D rotor geometry. Each geometry was then simulated to evaluate aerodynamic performance, focusing on turbine efficiency and reduced mass flow. This automated process ensured consistent model generation, minimized manual intervention, and allowed clear identification of performance trends for each geometric modification relative to the baseline rotor.

**Fig. 3:**
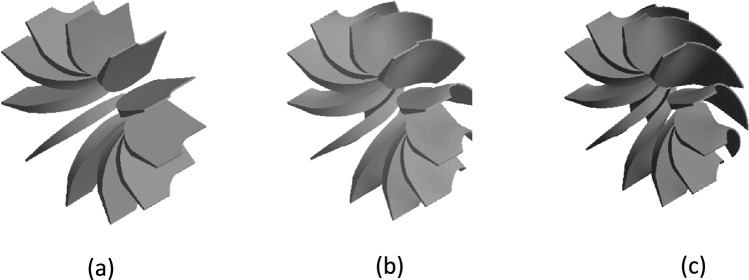
(**a**) Rotor with a decrease of 70% in blade angle distribution. (**b**) Baseline rotor. (**c**) Rotor with an increase of 50% in blade angle distribution.

To demonstrate the functionality of the automated rotor generator tool, several example geometries are presented. Figure 3 shows three rotors created by varying the blade angle distribution: the first represents the baseline, the second a higher angle, and the third the highest angle among the three. This illustrates how the tool can systematically modify a single geometric parameter while keeping all others constant, enabling direct assessment of the blade angle’s effect on rotor performance.

**Fig. 4:**
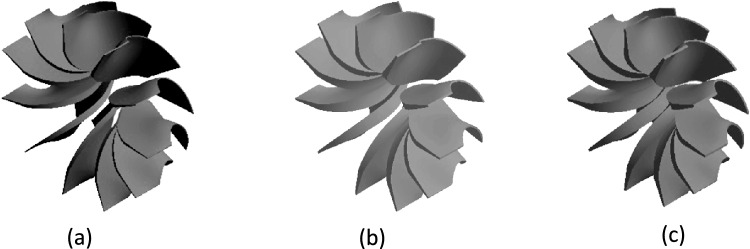
(**a**) Rotor with a decrease of 60% in blade thickness distribution. (**b**) Baseline rotor. (**c**) Rotor with an increase of 20% in blade thickness distribution.

Similarly, the automated tool can modify blade thickness distribution to generate rotors with different profiles. Figure 4 shows three geometries: the first with the lowest thickness, the second with moderate (baseline) thickness, and the third with the highest. This allows systematic analysis of how blade thickness affects aerodynamic performance, structural robustness, and flow behavior without manual re-modeling.

**Fig. 5:**
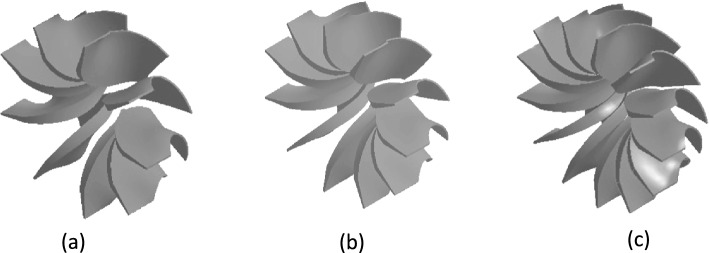
(**a**) Rotor with a decrease in blade count by 2 blades. (**b**) Baseline rotor. (**c**) Rotor with an increase in blade count by 2 blades.

Additionally, the tool allows easy variation of blade count to examine its effect on rotor performance. Figure 5 shows three geometries with 9, 11 (baseline), and 13 blades. By automatically updating the 3D geometry, the tool enables rapid generation of design variations, supporting efficient exploration of performance trends related to blade number.

In general, these examples illustrate the versatility and efficiency of the automated rotor generator tool. By allowing rapid, consistent, and reproducible modifications of key design parameters–blade angle, thickness, and count–the tool supports systematic parametric studies and provides a robust platform for optimizing rotor performance across multiple geometric dimensions.

## CFD and validation

### Computational setup and domain definition

**Fig. 6:**
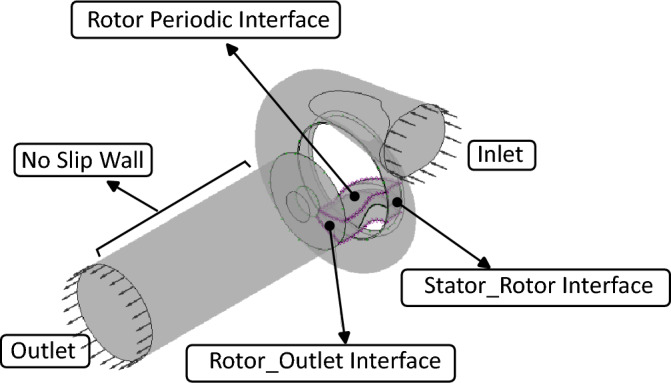
Computational setup and domain definition.

In the CFD modeling stage, the rotor design process began by importing the preliminary geometric dimensions obtained from the one-dimensional (1D) model. These baseline parameters were used to construct the rotor blades, applying the initial angle distribution and thickness profile to define the geometry. The computational domain was generated using a dedicated blade generator tool, ensuring accurate representation of the flow passages. The numerical setup included the required boundary conditions, as illustrated in Fig. 6, covering the rotor–stator interaction and flow outlet to capture realistic aerodynamic behavior.

The computational setup was divided into three domains: rotor, stator, and outlet. The rotor was defined as a rotating region, while the stator and outlet were stationary. The stator inlet was assigned ambient temperature and variable pressure to evaluate different expansion ratios, the outlet face ambient pressure, and all blade surfaces no-slip walls. Domain interactions were handled through fluid–fluid interfaces, with the rotor periodic interface exploiting symmetry to reduce computational cost without compromising accuracy.

For the steady-state CFD simulations, the interaction between the stator and rotor is modelled using the *mixing-plane* approach. In this method, flow variables at the stator–rotor interface are circumferentially averaged and passed to the rotor, effectively neglecting instantaneous circumferential fluctuations. This allows the rotor to be treated as periodically repeating, which simplifies the computational model and reduces the overall mesh size. Although the mixing-plane technique does not capture unsteady effects arising from the upstream volute or rotor–stator interactions, it remains an efficient and widely adopted method for examining aerodynamic trends associated with rotor geometry variations, including blade angle, thickness, and blade count^16–18^. To account for turbulence effects, the shear stress transport (SST) model was employed because of its robustness and proven reliability in turbomachinery simulations^19^. Finally, for the shroud surface–which follows the rotor geometry but remains physically stationary–a counter-rotating wall velocity was applied to ensure correct representation of relative motion in the simulation.

### Mesh sensitivity study

The meshing process was carried out with the Turbo Mesh module to create a high-quality grid suitable for turbomachinery simulations^20^. To ensure the reliability of the numerical results, a mesh sensitivity analysis was performed. This step is crucial because the accuracy of CFD predictions strongly depends on the grid resolution, especially in regions with high velocity gradients, boundary layers, and flow separations. Three different meshes with varying cell numbers and element sizes were generated and tested under the same boundary conditions. The results obtained from these grids were compared in terms of predicted performance parameters, such as efficiency and mass flow rate. Based on this comparison, the most suitable mesh was selected, providing a balance between computational accuracy and processing time, thereby ensuring reliable simulations without unnecessary computational expense.

**Fig. 7:**
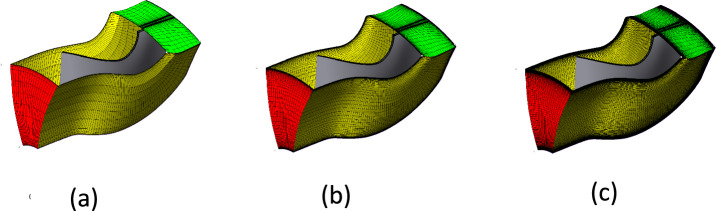
(**a**) Rotor mesh with 100k elements. (**b**) Rotor mesh with 300k elements. (**c**) Rotor mesh with 600k elements.

Figure 7 presents three representative mesh configurations for the rotor, where the computational domain was discretized using approximately 100,000, 300,000, and 600,000 elements. These meshes were generated to illustrate the effect of grid resolution on the accuracy of the numerical simulations, with the coarsest mesh providing faster computation at the expense of detail, while the finest mesh offers improved flow resolution around the rotor geometry.

To ensure grid-independent results, a comprehensive mesh convergence study was performed using six mesh densities: very coarse (60k), coarse (100k), medium (200k), fine (300k), very fine (500k), and ultra-fine (600k) elements. The variation in the predicted turbine performance (efficiency and pressure ratio) decreased progressively with mesh refinement, and convergence was achieved at approximately 300k elements. Beyond this level, the change in performance metrics was less than 0.1%, while the computational time increased by more than 3 h. This confirms that the 300k-element mesh provides an optimal balance between numerical accuracy and computational efficiency. Therefore, this mesh was adopted for all subsequent CFD simulations. The results of this mesh convergence study are summarized in Table 1.

**Table 1:** Summary of mesh convergence study results.

Mesh size (elements)	Efficiency (%)	RMF	Computation time (h)
60k	78.1	0.889	2
100k	78.7	0.897	2.75
200k	79.1	0.910	3.5
300k	79.3	0.912	5
500k	79.38	0.913	7
600k	79.41	0.9135	8

### Validation of CFD predictions

To ensure the reliability of the numerical predictions, the CFD results of the radial turbine rotor were validated against experimental data obtained from the BorgWarner Turbo Systems K03/05 turbocharger^13^. The experiments were conducted on the hot gas test facility at the Institute of Thermal Turbomachinery (ITSM), University of Stuttgart, which offers well-controlled boundary conditions and high measurement accuracy. Key performance parameters, including turbine efficiency and mass flow characteristics, were compared between simulation and experiment. The *Reduced Mass Flow Rate* (RMF), expressed in units of $\mathrm {kg \,s^{-1} \,K^{0.5} \,bar^{-1}}$, was used as a normalized metric to account for variations in gas density and temperature, enabling consistent comparison of mass flow behavior across different operating conditions. The numerical results showed good agreement with the experimental measurements, capturing the overall performance trends and deviations within acceptable limits. This validation confirms the credibility of the CFD model and its capability to predict the aerodynamic behavior of the turbine rotor under realistic operating conditions.

**Fig. 8:**
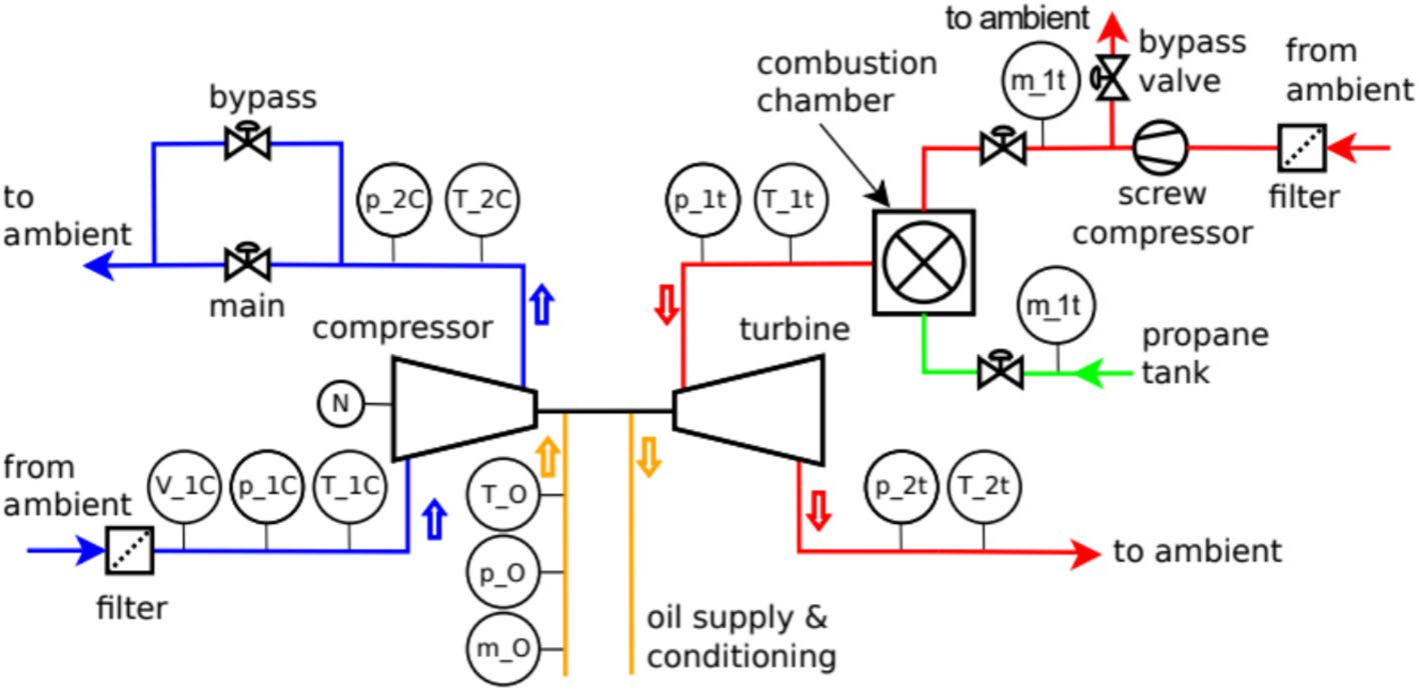
Schematic of the hot gas test rig used for rotor experiments, showing the main components and flow path of the working gas.

Figure 8 shows a schematic of the hot gas test rig used for the radial turbine rotor experiments. The setup consists of a screw compressor supplying air to the combustion chamber, where the working gas is heated before entering the turbine. The diagram highlights the main components–including the compressor, combustion chamber, turbine, measurement ports, and bypass lines–and indicates the flow direction of the hot gas. This layout enables controlled testing of the rotor’s total-to-static efficiency and mass flow characteristics, with the measurement ports providing critical data for performance evaluation.

**Fig. 9:**
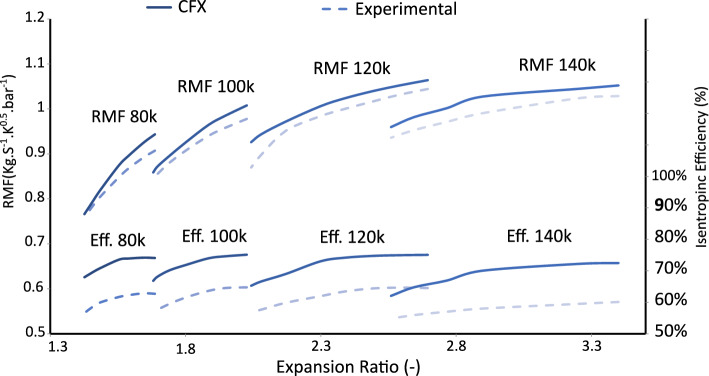
Validation of CFD predictions against experimental measurements: (**a**) total-to-static efficiency and (**b**) reduced mass flow (RMF) for the radial turbine rotor. Reproduced from Fawaz, M.A.; Ayad, A.F.; Shaheen, M.; Mahmoud, H.; Al-samieh, M.F., Research Square preprint, 2024, DOI: 10.21203/rs.3.rs-7663106/v1, licensed under CC BY 4.0^15^.

Figure 9 compares the CFD-predicted and experimentally measured performance of the radial turbine at four rotational speeds–80,000, 100,000, 120,000, and 140,000 rpm–across a range of expansion ratios from 1.5 to 3. For each speed, a set of cases was constructed to evaluate both the isentropic efficiency and the reduced mass flow (RMF), providing a comprehensive comparison between numerical and experimental trends. During turbocharger turbine testing, experimentally measured efficiencies often fall short of CFD predictions due to unavoidable thermal losses through metal components. CFD simulations typically assume perfectly adiabatic conditions, neglecting these losses and thus overestimating efficiency. Depending on gas temperature, insulation quality, and operating load, such thermal losses may account for 5–10% of the gas power, reducing the measured turbine efficiency by roughly 3–8 percentage points. Nevertheless, these experimental values are supported by well-established studies^16–18^, which provide comprehensive analyses of radial turbines with multi-channel casing designs and serve as reliable references for validating CFD results. The comparison demonstrates that, despite the thermal discrepancies, the numerical predictions accurately capture the key performance characteristics of the turbine, reinforcing the credibility of the CFD approach and confirming its applicability for further analysis and design optimization. Consequently, the observed deviation does not compromise the validity of the conclusions regarding the effects of blade geometry on rotor performance.

## Results

In this section, the results of varying the blade angle distribution, blade thickness distribution, and the number of blades on turbine performance are presented and discussed. The analysis focuses on their influence on efficiency and reduced mass flow rate, highlighting the contribution of each geometric parameter to overall rotor behavior. Detailed flow-field visualizations are provided to illustrate the aerodynamic mechanisms behind these performance variations. Through this analysis, the reasons for performance improvements or degradations are identified, offering deeper insight into how blade geometry governs internal flow structures and energy conversion efficiency.

### Effect of blade angle distribution on rotor performance

**Fig. 10:**
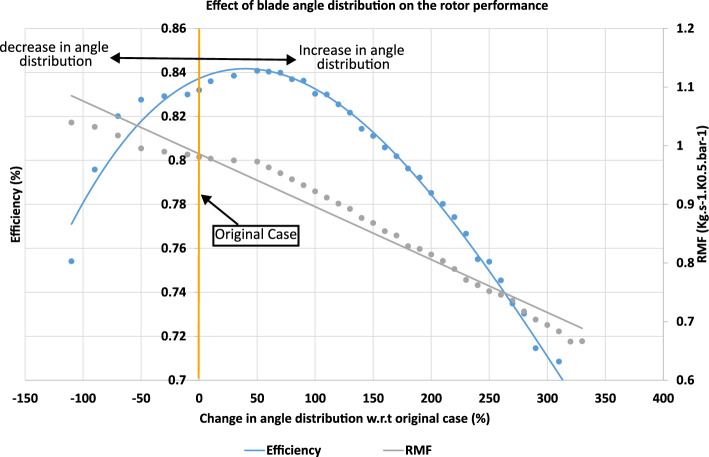
Effect of rotor blade angle distribution on turbine performance.

Starting from the original rotor configuration, different cases were constructed by systematically increasing and decreasing the blade angle distributions of the camberlines by fixed percentages of their original values. For each case, the turbine efficiency and reduced mass flow rate were evaluated, collected, and plotted as illustrated in Fig. 10 to determine the governing relationships between angle distribution and performance.

The results indicate that efficiency increases with blade angle distribution up to a peak value, after which it decreases, a trend well represented by a third-order polynomial equation (Eq. 1). In contrast, the reduced mass flow rate exhibits a nearly linear decrease with increasing angle distribution (Eq. 2), which can be attributed to the reduction of throat area. For instance, increasing the blade angle by 50% resulted in an improvement of the isentropic efficiency from 83.2% to 84.1%, while the reduced mass flow decreased slightly from 0.982 to 0.981, illustrating the quantitative impact of blade angle changes on rotor performance.

1 $$\begin{aligned} \eta _{is}= & 3\times 10^{-9}x^{3} - 3\times 10^{-6}x^{2} + 0.0002x + 0.8372 \end{aligned}$$

2 $$\begin{aligned} RMF= & -0.0009x + 0.986 \end{aligned}$$

$$\eta _{is}$$ ...Isentropic efficiency.

*RMF*, Reduced mass flow rate; *x*, Change in angle distribution relative to the original case (%).

**Fig. 11:**
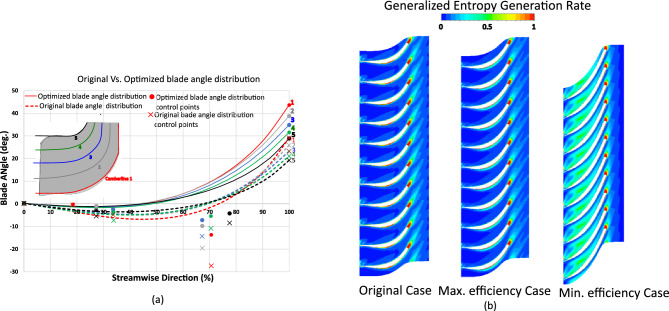
(**a**) Blade angle distribution for the original and maximum-efficiency cases. (**b**) Entropy generation rate comparison of the original, maximum-, and minimum-efficiency cases due to blade angle effect.

To illustrate the effect of geometry, the blade angle distributions of the five camberlines for both the original and maximum-efficiency cases were plotted as Bézier curves with their control points, where camberline 1 corresponds to the hub and camberline 5 to the shroud (Fig. 11a). Control points define the shape of a Bézier curve: the curve passes through the first and last points, while the other points determine its overall curvature, allowing precise adjustment of the blade shape.

The entropy generation rate at 0.3 span was visualized for the original, maximum-efficiency, and minimum-efficiency cases, as illustrated in Fig. 11b, to clarify the aerodynamic mechanisms driving efficiency changes. The original rotor exhibits pronounced dissipation near the suction side, blade tip, and hub due to secondary flows, wake interactions, and tip-leakage vortices. In contrast, the optimized configurations show a marked reduction in these high-entropy regions, particularly near the leading edge and shroud, indicating improved flow alignment and weaker secondary structures. The design maintaining the original RMF achieves moderate entropy reduction around the mid-chord, while the maximum-efficiency configuration shows substantial reduction across the entire blade passage, confirming that optimization effectively mitigates local flow separation and secondary losses.

**Fig. 12:**
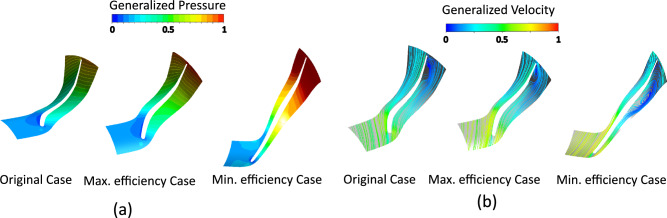
(**a**) Pressure contours (**b**) Velocity streamlines comparison between original, maximum-, and minimum-efficiency cases due to blade angle effect.

Comparative analysis of pressure contours and velocity streamlines (Fig. 12) further supports these observations, showing how optimized blade angle distributions improve flow guidance, reduce losses, and increase efficiency relative to the original rotor.

### Effect of blade thickness distribution on rotor performance

**Fig. 13:**
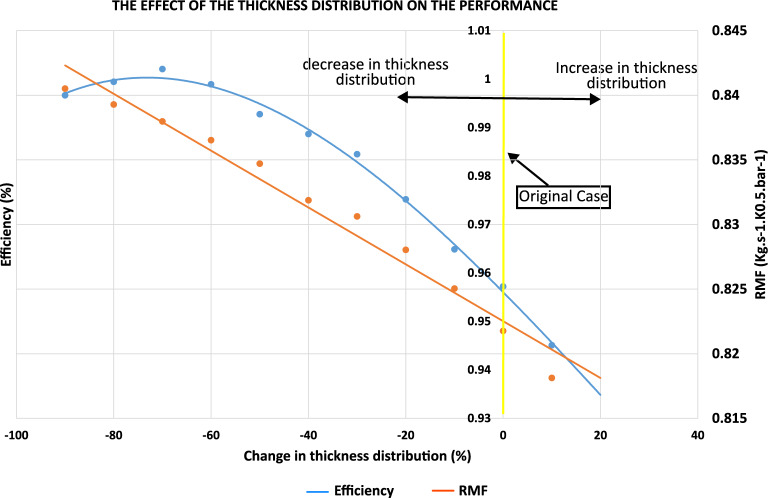
Effect of rotor blade thickness distribution on turbine performance.

Starting from the original rotor configuration, cases were generated by systematically varying the blade thickness distributions of the camberlines by fixed percentages of their original values. The results show that efficiency initially increases with decreasing thickness until reaching a peak, after which it declines, following a third-order polynomial trend (Eq. 3). Meanwhile, the reduced mass flow rate decreases almost linearly with increasing thickness (Eq. 4) due to reduction of effective flow passage, as illustrated in Fig. 13.

3 $$\begin{aligned} \eta _{is}= & 1\times 10^{-8}x^{3} - 1\times 10^{-6}x^{2} - 0.0004x + 0.8248 \end{aligned}$$

4 $$\begin{aligned} RMF= & -0.0006x + 0.9501 \end{aligned}$$

**Fig. 14:**
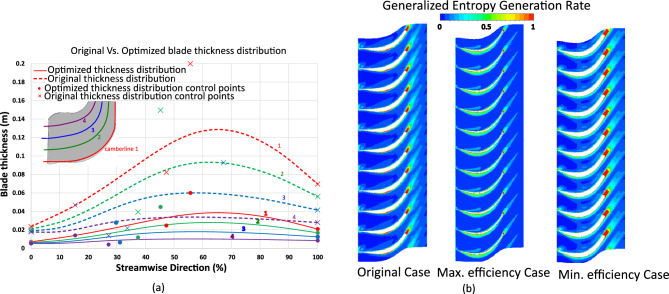
(**a**) Blade thickness distribution for the original and maximum-efficiency cases. (**b**) Entropy generation rate comparison of the original, maximum-, and minimum-efficiency cases due to thickness effect.

Figure 14a shows the thickness distributions of the five camberlines for the original and maximum-efficiency cases as Bézier curves.

Figure 14b shows the radial distribution of entropy generation for different thicknesses. Optimal thickness reduces entropy near the hub and inlet, indicating lower irreversibilities, while thicker or thinner blades increase local losses. Reducing trailing edge thickness and smoothing curvature minimizes wake mixing losses, and optimizing the leading edge geometry improves incidence angle, reducing shock-induced and separation-related irreversibilities, thus enhancing flow guidance and rotor efficiency.

**Fig. 15:**
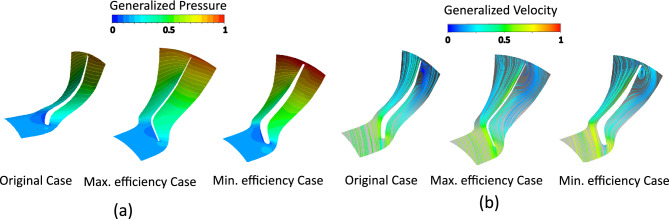
(**a**) Pressure contours. (**b**) Velocity streamlines comparison between original, maximum-, and minimum-efficiency cases due to thickness effect.

Figure 15 presents static pressure contours and velocity streamlines for different thicknesses. Optimized thickness produces smoother pressure gradients, weaker vortices, reduced tip leakage, and cleaner wakes, leading to better flow guidance and higher efficiency.

### Effect of number of blades on rotor performance

**Fig. 16:**
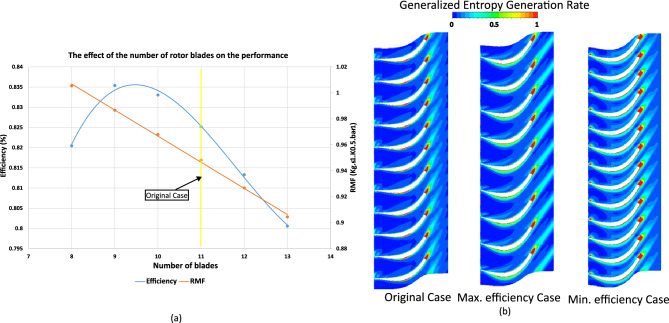
(**a**) Effect of rotor blade number on turbine performance. (**b**) Entropy generation rate comparison of the original, maximum-, and minimum-efficiency cases due to blade number effect.

Starting from the original rotor configuration, cases were generated by systematically varying the number of blades. Efficiency initially increases with blade number until reaching a maximum, after which it declines, following a third-order polynomial trend (Eq. 5). Meanwhile, the reduced mass flow rate decreases almost linearly with increasing blade number (Eq. 6) due to reduction of effective flow passage, as illustrated in Fig. 16a.

Figure 16b compares the radial entropy generation rate for original, maximum-, and minimum-efficiency cases. The maximum-efficiency case shows reduced entropy near the hub and inlet, indicating lower irreversibilities, while the minimum-efficiency case exhibits higher entropy. Proper blade count balances solidity, reducing blockage and frictional losses, resulting in smoother flow turning, weaker vortices, and reduced wake interactions.

**Fig. 17:**
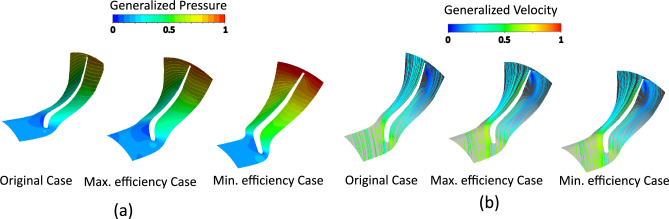
(**a**) Pressure contours. (**b**) Velocity streamlines comparison between original, maximum-, and minimum-efficiency cases due to blade count.

Figure 17 compares pressure contours and velocity streamlines for different blade counts. The maximum-efficiency case shows smoother pressure gradients, attached suction-side flow, weaker cross-passage forces, reduced tip leakage, and cleaner wakes. Streamlines are evenly distributed with minimal jet–wake formation, whereas the minimum-efficiency case exhibits stronger pressure gradients, vortices, and flow separation.

5 $$\begin{aligned} \eta _{is}= & 0.0008N^{3} - 0.0284N^{2} + 0.3225N - 0.351 \end{aligned}$$

6 $$\begin{aligned} RMF= & -0.0201N + 1.1678 \end{aligned}$$

*N*, Rotor number of blades.

## Discussion

The present investigation provides a systematic evaluation of how three fundamental geometric parameters–blade angle distribution, thickness profile, and blade count–affect the aerodynamic performance of radial turbine rotors. By employing an automated design tool coupled with high-fidelity CFD simulations, it was possible to isolate the contribution of each parameter and assess their separate implications for turbine efficiency, reduced mass flow, and internal flow structures. The trends observed across the parametric variations shed light on the delicate balance between aerodynamic loading, flow guidance, and loss mechanisms within the rotor passage.

The variation of blade angle distribution revealed a clear efficiency peak at an intermediate configuration, beyond which further increases led to performance deterioration. This behavior can be attributed to the competing influence of flow alignment and throat area reduction. Moderate angle adjustments improved flow turning and reduced separation near the hub and shroud, as demonstrated by the entropy generation maps, while excessive angles constrained the effective flow passage, raising incidence and accelerating losses. These findings reinforce the notion that angle optimization must balance aerodynamic benefits with geometric constraints, particularly throat blockage effects.

For blade thickness distribution, reducing thickness to an optimal level enhanced efficiency by mitigating wake mixing and shock-related irreversibilities. Thinner trailing edges produced cleaner exit wakes and more uniform velocity distributions, while overly thin sections risked increased incidence losses and possible structural compromises. Conversely, excessively thick blades introduced additional blockage and adverse pressure gradients, which encouraged boundary layer separation and secondary vortex formation. The results suggest that thickness profiling should be tailored to balance aerodynamic performance with mechanical reliability.

Blade count variations also exhibited a non-linear relationship with efficiency. A moderate increase in the number of blades improved flow guidance and reduced cross-passage deviations, leading to more favorable pressure and velocity fields. However, beyond the optimal count, frictional losses and excessive solidity counteracted these benefits, as evidenced by the higher entropy generation in dense-blade configurations. The reduced mass flow rate observed with higher blade counts highlights another practical trade-off: while additional blades can smooth flow guidance, they simultaneously restrict the flow passage and increase viscous losses. The optimal blade number thus emerges as a compromise between maintaining effective aerodynamic control and avoiding excessive blockage.

Taken together, these results emphasize that rotor performance cannot be optimized by focusing on a single parameter in isolation. Blade angle, thickness, and count interact to shape the internal flow structure, and each parameter exhibits diminishing returns beyond a certain threshold. The automated rotor generation tool proved effective in capturing these trends, enabling consistent geometric modifications and direct comparison of performance outcomes. Importantly, the results underline the value of entropy generation analysis in diagnosing loss mechanisms, providing physical explanations for efficiency trends beyond global performance metrics.

### Comparison with prior studies and novelty

Previous studies have examined individual geometric effects on radial turbines–such as blade angle distribution^21^, blade thickness profiles^4^, and blade count^7^–but generally focused on limited ranges, often considered stator or multistage configurations, and did not systematically isolate the effect of each parameter. In contrast, the present study investigates rotor geometry exclusively, analyzing the effect of each parameter separately to clearly describe its influence on performance. The parameter ranges explored are: blade angle distribution from -90% to +100% relative to baseline, blade thickness from 0.1 to 1.3 times the baseline values, and blade count from 7 to 14 blades.

By isolating each parameter, the study reveals trends in efficiency, reduced mass flow, flow guidance, and entropy generation that are not apparent when parameters are combined or explored in limited ranges. Compared to prior studies (Table A1, Supplementary material), the current work offers broader parameter coverage, detailed flow-field analysis, and deeper physical insight, establishing its novelty in quantifying individual rotor geometric effects.

The optimized blade angle, thickness, and count improve flow guidance, suppress separation and secondary losses, and produce more uniform exit conditions, enhancing overall turbocharger and engine performance. Efficiency gains are achieved with moderate increases in aerodynamic load, maintaining acceptable operational limits and demonstrating a balanced trade-off between performance and rotor reliability.

Overall, careful geometric tailoring–moderate increases in angle and blade count combined with strategically thinned profiles–yields measurable efficiency improvements without compromising mass flow or mechanical loading, providing practical guidance for high-efficiency, application-specific radial turbine rotor design.

### Practical implications and limitations

The aerodynamic trends identified in this investigation translate into several practical benefits for real turbine operation. Adjustments in blade angle, thickness profile, and blade count lead to improved flow alignment through the rotor, lower separation levels, and more uniform exit conditions, all of which contribute to higher total-to-static efficiency. In turbocharger applications, however, the turbine and compressor operate at a single matched point, and the power balance between both components determines the operating condition^22,23^. As a result, any aerodynamic enhancement inherently influences the loading level required to supply the compressor demand. The geometries that exhibited increased efficiency in this study are therefore accompanied by corresponding shifts in aerodynamic load, reflected in pressure distribution and torque generation across the rotor. These variations remain moderate within the explored parameter space and do not indicate critical deviations from typical operational loading ranges^4^.

It must be noted that the present analysis examines only the aerodynamic aspects of rotor performance. While thinner blades or higher blade counts can offer beneficial efficiency gains, such changes also modify mechanical loading–particularly centrifugal and bending stresses–which were not assessed in this work. Prior research has shown that geometric modifications affecting aerodynamic loading can have significant structural implications, especially for high-speed radial turbines^7,13^. Because excessive thinning or high solidity may reduce structural safety margins, a complete design evaluation should incorporate coupled aerodynamic–structural methods. Future work should integrate CFD-based flow analysis (e.g., ANSYS CFX^10^) with finite-element stress assessment to ensure that performance improvements do not compromise long-term rotor durability.

Overall, the results highlight clear sensitivities and practical trade-offs for blade angle, thickness, and count, showing that carefully controlled geometric adjustments can improve efficiency while maintaining mass flow and aerodynamic loading within acceptable limits. These insights support the design and optimization of high-efficiency radial turbine rotors for turbochargers, small-scale energy systems, and propulsion applications.

## Conclusion

This study investigated the effects of blade angle, blade thickness, and blade count on the aerodynamic performance of a radial turbine rotor using an automated rotor generation tool coupled with high-fidelity CFD simulations. The main findings are:

- *Blade angle:* Efficiency increases up to an optimal angle, beyond which blockage and incidence losses reduce performance; mass flow decreases nearly linearly with increasing angle.
- *Blade thickness:* Moderate thinning improves efficiency by reducing separation and secondary losses; excessive thinning can raise incidence losses and affect structural integrity. Mass flow decreases with thicker blades.
- *Blade count:* Adding blades initially enhances efficiency through better flow guidance and reduced entropy generation; beyond the optimum, friction and blockage degrade performance. Mass flow decreases almost linearly with blade number.
- *Performance trends:* Efficiency exhibits non-linear behavior, while reduced mass flow shows near-linear dependence on geometric changes.
- *Flow mechanisms:* Optimal configurations suppress separation, weaken secondary vortices, and improve aerodynamic uniformity, as seen from entropy, velocity, and pressure fields.
- *Design implications:* The study identifies first-order sensitivities and trade-offs, providing guidance for improving efficiency, maintaining mass flow, and controlling aerodynamic loading.

Overall, the results demonstrate that a systematic, physics-based approach effectively quantifies the influence of key geometric parameters on radial turbine performance and supports future design and optimization efforts.

## Supplementary Information

Supplementary Information.

## Data Availability

The datasets used and/or analysed during the current study are available from the corresponding author, Mostafa Abdo Fawaz, on reasonable request.
