# Supplementary material for: Effect of blade parameters on radial turbine rotor aerodynamics
Source: Sci Rep. 2026 Jan 14;16:2070. doi: 10.1038/s41598-025-33442-4 (PMC12808112; doi:10.1038/s41598-025-33442-4)
Supplement: Supplementary file 1 — Supplementary Information. [file 41598_2025_33442_MOESM1_ESM.pdf]

Appendix A Comparison of the present work with selected prior studies on radial turbines.

Table A1 Comparison of the present work with selected prior studies on radial turbines.

**Study**	**Parameter(s) varied**	**Operating envelope**	**Key findings**	**Comparison with present work**
Li et al. (2018) [?]	Blade installation angle (stator/rotor)	32° angle	Flow losses minimized at 32°	We explore rotor angle variation only, over a broader range (-90% to +100% w.r.t baseline), and evaluate impact on efficiency and reduced mass flow.
Schobeiri et al. (2005) [?]	Blade thickness profile	Tail-edge/throat ratios typical of industrial rotors	Thickness affects separation and wake losses	We study rotor thickness distribution across full span (0.1–1.3× baseline) and quantify effects on entropy generation, flow separation, and efficiency.
Kim (2016) [?]	Blade count	9–13 blades	Trade-off between guidance and friction	Rotor blade count systematically varied from 7 to 14, linking flow uniformity, entropy generation, and efficiency to blade number.
**Present study**	Blade angle distribution, thickness distribution, blade count (each separately)	Angle: -90% to +100%, Thickness: 0.1–1.3× baseline, Blade count: 7–14	Identified optimal configurations for each parameter, mapped effects on flow, entropy generation, and performance	Novel in providing a detailed parametric study of each parameter separately for the rotor, with broader ranges and physical flow analysis.
